# Acro-Ischemia Associated With SARS-CoV-2: A Case Report

**DOI:** 10.7759/cureus.53798

**Published:** 2024-02-07

**Authors:** Jesus Ivan Martinez-Ortega, Felipe de Jesus Perez Hernandez, Angel Enrique Ortegon Blanco

**Affiliations:** 1 Department of Dermatology, Instituto Dermatológico de Jalisco "Dr. José Barba Rubio", Zapopan, MEX; 2 Department of Internal Medicine, Hospital Regional de Alta Especialidad de la Península de Yucatán, Merida, MEX; 3 Department of Internal Medicine, Instituto de Seguridad y Servicios Sociales de los Trabajadores del Estado, Clínica Hospital B, Chetumal, MEX

**Keywords:** sars-cov-2, treatment option, covid 19, anticoagulation, acroischemia

## Abstract

COVID-19 is known to cause various cutaneous lesions, including acro-ischemic lesions (AIL), which are associated with poor prognosis. Anticoagulant therapy has shown positive responses in AIL patients. However, in this case study, we present a fatal AIL case despite anticoagulant therapy. We propose different treatment approaches based on the limited current data on acro-ischemia pathogenesis related to SARS-CoV-2. The clinical case involved a 59-year-old male with severe COVID-19 symptoms, including acrocyanosis and right hemiparesis. Despite receiving anticoagulant therapy, the patient's condition worsened, leading to necrosis in the left foot. The discussion focuses on the high-risk nature of AIL, the potential link between angiotensin-converting enzyme 2 (ACE2) receptors and vasculitis or thromboembolic manifestations, and the role of immune clots in AIL pathogenesis. Behçet syndrome is referenced as a model of inflammation-induced thrombosis, guiding the suggestion for immunosuppressant-based treatment in addition to anticoagulants. Additionally, three substances, N-acetyl cysteine, sulodexide, and hydroxychloroquine, are proposed.

## Introduction

COVID-19 can lead to various cutaneous lesions, including maculopapular rash, perniosis, livedo reticularis, multisystem inflammatory syndrome, and acro-ischemic lesions (AIL). AIL is caused by secondary microthrombosis caused by endothelial damage and vascular disorders [1]. AIL can affect infants and adults with mild or severe COVID-19 symptoms. Some studies have suggested that AIL may present a poor prognosis [2]. SARS-CoV-2 enters the cells via angiotensin-converting enzyme 2 (ACE2). ACE2 receptors are expressed in lung epithelial cells, macrophages, enterocytes, and other cells. The activation of these receptors leads to an inflammatory immune response that may be responsible for lung and other organ damage associated with the virus [3]. Different studies have suggested anticoagulant therapy in patients with AIL [4].

In this study, we present an AIL case that showed COVID-19 respiratory and systemic symptoms, which culminated in necrosis and fatality despite the use of anticoagulant therapy. Thus, we proposed different treatment approaches based on the scarce current data on acro ischemia pathogenesis related to SARS-CoV-2.

This article was previously presented as a poster abstract in 2023 at the World Congress of Dermatology.

## Case presentation

A 59-year-old male presented to the emergency room in February 2021, complaining about fever, odynophagia, and shortness of breath. Physical examination at admission revealed oxygen saturation of 85%, decreased alertness, tachycardia of 114 beats per minute, tachypnea of 24 per minute, temperature of 37.3° C, and normal blood pressure (100/70mmHg). He also showed acrocyanosis in the feet and right hemiparesis. His vital signs and oxygen levels improved after treatment with a face mask; however, his neurological status did not improve.

A polymerase chain reaction (PCR) test for SARS-CoV-2 on a nasopharyngeal swab sample was positive; therefore, according to severity, dexamethasone 6 mg once a day was started. Due to the persistence of decreased neurological status, a head computed tomography (CT) was conducted, which revealed thalamic and pontomesencephalic hypodensities with hemorrhagic conversion. A CT of the chest revealed peripheral ground-glass opacities and consolidation at the lungs. EKG didn't reveal any abnormalities.

**Figure 1:**
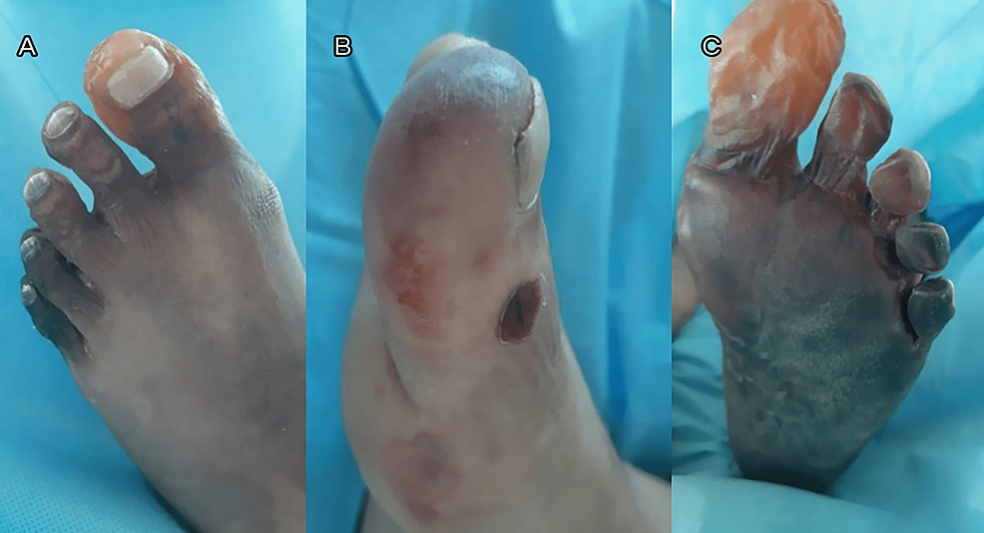
Clinical images of acro-ischemic lesions A, B: left and right foot at admission, respectively; C: left foot at a later evaluation

During his stay, cyanosis of the left foot progressed to necrosis (see Figure 1A-B).

The neurological status of the patient worsened, and he received mechanical ventilation.

Laboratory studies revealed high D-dimer (5.1 mg/ml), C-reactive protein (8.5 mg/dl), and ferritin (699 ng/ml) levels. Despite adequate pulmonary evolution, necrosis persisted in the left foot, as seen in Figure 1C.

Doppler ultrasonography did not identify stenosis or clots, and an echocardiography ruled out cardiac or valvular involvement.

**Figure 2:**
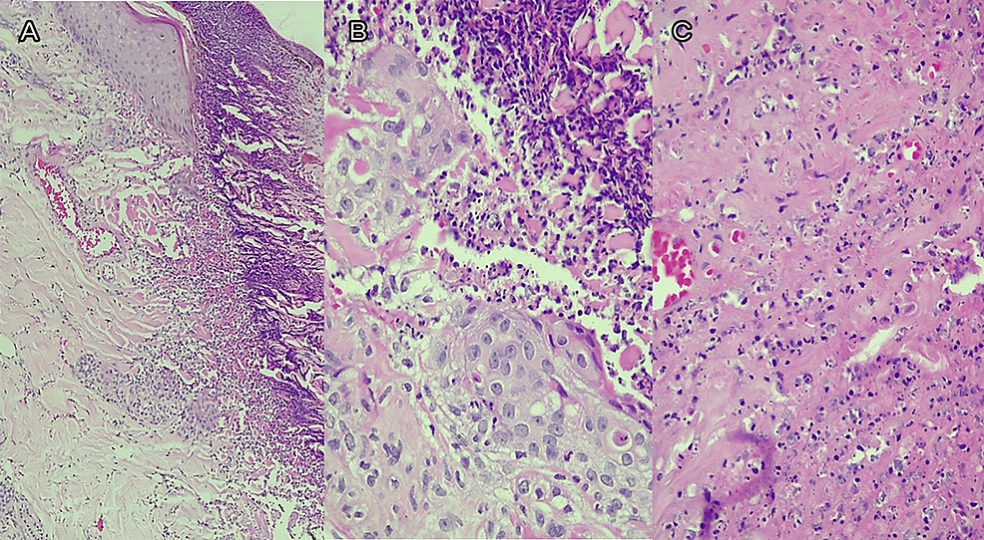
Skin biopsy with hematoxylin and eosin stain Skin biopsy of necrotic area with hematoxylin and eosin staining showing leukocytoclastic vasculitis. A: necrosis of the whole thickness of the epidermis (10x magnification); B: perivascular inflammatory infiltrate (40x magnification); C: significant fibrin deposits and nuclear debris (40x magnification)

Histopathology showed small vessel vasculitis and necrotic epidermis with perivascular pauci-immune infiltrates (Figure 2).

The patient received anticoagulation with low molecular weight heparin (LMWH) and platelet antiaggregating therapy without improvement. Due to the extension of necrosis of the left foot, the patient underwent supracondylar amputation. Unfortunately, after three days, the patient died.

It is important to mention that by the time of the patient's admission, there were no specific antivirals for SARS-CoV-2 with clinical evidence of effectiveness in severe cases, and although there were some in different clinical phases, they were not available in our hospital.

## Discussion

Acro-ischemia, observed in the initial cases of COVID-19 reported by Zhang et al. in Wuhan, highlighted a novel cutaneous manifestation linked to SARS-CoV-2 during the pandemic's onset. Notably, their findings suggested a potential association between acro-ischemia and COVID-19, prompting consideration for anticoagulant therapy as a management strategy for the disease's dermatological symptoms [4]. Subsequently, a Spanish research group classified acro-ischemia manifestations in 377 patients, raising concerns about their prognostic implications and the possibility of indicating a more severe disease course [5].

Moreover, Attisani et al.'s systematic review specifically focused on distal ischemia associated with COVID-19. Through an analysis of 36 articles encompassing 194 patients, the review outlined the high risk of revascularization failure and perioperative mortality in individuals presenting with this condition [6].

Among 344 Hispanic patients, distal manifestations of ischemia were observed in 6% of the cases [7].

Recent studies have highlighted that platelets harbor the cyclic guanosine monophosphate-adenosine monophosphate (GMP-AMP) synthase (or cGAS) and stimulator of interferon genes (STING) proteins, functioning as cytoplasmic receptors in viral infections, potentially contributing to platelet activation and aggregation. However, it appears that there are additional components involved in this pathway, which we will endeavor to discuss [8].

SARS-CoV-2 enters cells through the ACE2 receptor, which is expressed in endothelial cells. These receptors make endothelial cells susceptible to vasculitis, endothelitis, and vasculitic manifestations. The patient's response, whether more balanced towards an interferon (IFN) response or proinflammatory cytokines like tumoral necrosis factor (TNF), determines the prevalence of vasculitic or thromboembolic manifestations, respectively [9].

Moreover, ACE2 receptors are also expressed in alveolar cells, leading to SARS-CoV-2 affecting the alveoli and peripheral areas of the lungs, unlike influenza and other respiratory viruses that primarily target bronchioles [9]. The proinflammatory mediators produced in the alveoli, along with RNAemia, enter the capillary network and target endothelial cells, causing the production of proinflammatory and procoagulant mediators, ultimately leading to immunothrombosis. These immune clots differ from "normal" clots, mainly comprising fibrin, as they consist of abundant immune cells, particularly neutrophils. These immune clots, often located at anastomoses and connections between arterial and venous vessels, are prone to form emboli that can travel to distant sites through the heart's cavities, eventually reaching the smallest capillaries in the skin [9-11]. Neutrophils and NETosis contribute to their highly procoagulant nature, leading to further clot growth [12]. 

Behçet syndrome serves as a natural model of inflammation-induced thrombosis, driven by neutrophil hyperactivation and neutrophil-mediated mechanisms that result in platelet activation and thrombogenesis [13]. Thus, the treatment approach for acro-ischemia should focus more on immunosuppressants rather than anticoagulants, drawing insights from this model. In addition to anticoagulants, we propose integrating the limited data on acro-ischemic pathogenesis and incorporating anti-inflammatory treatments, including steroids. Furthermore, we consider two promising substances: N-acetyl cysteine and sulodexide. These compounds show promise for their potential suitability based on their multifaceted mechanisms, including antioxidant, vascular, and anti-inflammatory effects. Sulodexide, a natural glycosaminoglycan, exhibits a diverse range of benefits, encompassing antithrombotic and profibrinolytic effects, positive hemorheological impact, and attenuation of ischemia-reperfusion injury [14]. Meanwhile, N-acetyl cysteine intervenes in the vitamin K reduction pathway and acts as a precursor to the antioxidant glutathione, suggesting its potential in both antioxidant and anti-inflammatory capacities [15].

Another intriguing pathogenic mechanism, which may be related, is the syncytium formation; it should be noted that these mechanisms are not mutually exclusive, as they might operate simultaneously or at different times in the pathogenesis of COVID-related ischemia. The cleavage of the bi-arginine motif releases the 4-amino acid sequence (PRRA) from the spike S glycoprotein of SARS-CoV-2. This sequence is responsible for inducing syncytium formation, a process that has been implicated in SARS-CoV-2-associated thrombosis. It has been hypothesized that syncytium formation could lead to the assembly of tissue factor, factor VIIa, and externalized membrane-associated phosphatidylserine, initiating the coagulation cascade. However, for this hypothesis to hold true, syncytium exposure to blood might be necessary [16]. Thus, we propose that in the aforementioned pathogenesis model, the syncytium formation of immune cell infiltrates could easily access the capillaries associated with the alveoli. Interestingly, distant thrombosis sites could be explained by emboli composed of immunoclot, potentially containing syncytia, along with other mechanisms (such as neutrophil extracellular trap, NET). We believe that the cleaved PRRA sequence from the spike S glycoprotein of SARS-CoV-2 could travel to distant locations through the bloodstream.

**Figure 3:**
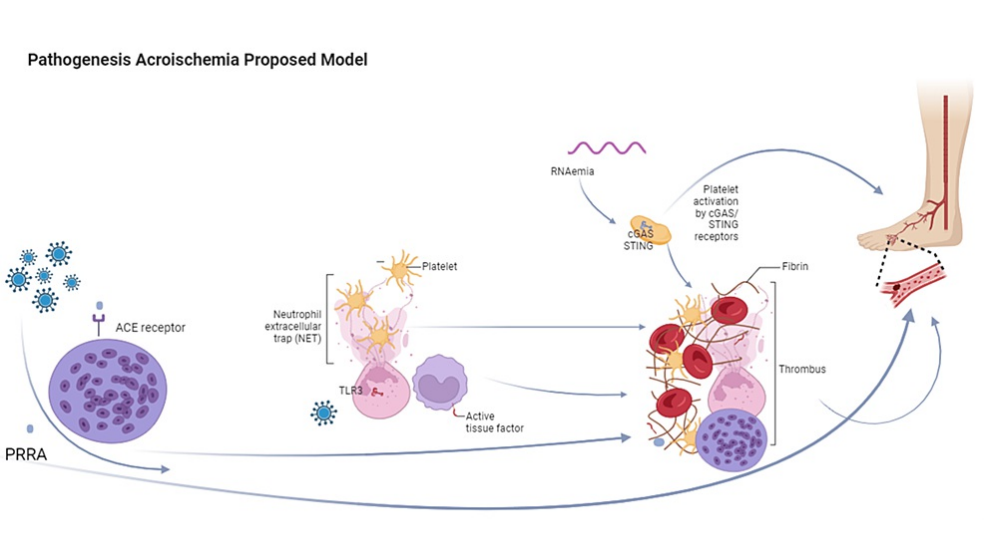
Proposed model of acro-ischemia pathogenesis The illustration shows on the left side the formation of syncytia induced by the PRRA motif from the spike protein. This process may contribute to the formation of immunothrombosis and distant emboli, or the PRRA motif may independently induce distant thrombosis. In the middle, the ssRNA is shown to activate the intracellular receptor TLR3 on neutrophils, triggering NETosis. This process involves the entrapment of platelets and procoagulant mediators, ultimately leading to the formation of immunothrombosis, as depicted on the right. Additionally, platelets may be activated by cGAS-STING intracellular receptors, further contributing to immunothrombosis. ssRNA - single-stranded RNA; TLR3 - toll-like receptor 3; cGAS - the cyclic guanosine monophosphate–adenosine monophosphate (GMP-AMP) synthase; ​​​​​​STING - stimulator of interferon genes The image is created with biorender.com.

Thus, either the presence of syncytia within the immunoclot or the release of the PRRA sequence from the spike S protein could potentially explain the pathogenesis of acro ischemia. Notably, hydroxychloroquine has demonstrated potent inhibition of S glycoprotein processing and membrane fusion [16]. Interestingly, hydroxychloroquine has also been shown to inhibit NET formation [17]. As a summary of the above, we propose an adapted pathogenic model in Figure 3.

These substances could play a crucial role in addressing the complex pathogenesis of acro-ischemia associated with SARS-CoV-2 infection. Targeting these specific mechanisms may offer valuable therapeutic options to complement the standard anticoagulant treatments and potentially improve patient outcomes.

However, further research is needed to better understand their potential role in the context of acro-ischemia related to COVID-19.

## Conclusions

Acro-ischemic lesions associated with COVID-19 present a complex clinical challenge with potentially severe outcomes. Anticoagulant therapy has shown positive responses in some cases, but AIL can still progress despite standard treatment. Considering the multifaceted pathogenesis, a combined approach using immunosuppressants alongside anticoagulants may be beneficial. Further research is needed to optimize treatment and improve outcomes for AIL associated with SARS-CoV-2 infection.

## References

[1] Fernández-Lázaro D, Garrosa M (2021). Identification, mechanism, and treatment of skin lesions in COVID-19: a review. Viruses.

[2] Putko RM, Bedrin MD, Clark DM, Piscoya AS, Dunn JC, Nesti LJ (2021). SARS-CoV-2 and limb ischemia: a systematic review. J Clin Orthop Trauma.

[3] Ni W, Yang X, Yang D (2020). Role of angiotensin-converting enzyme 2 (ACE2) in COVID-19. Crit Care.

[4] Zhang Y, Li H, Zhang J (2020). The clinical characteristics and outcomes of diabetes mellitus and secondary hyperglycaemia patients with coronavirus. Diabetes Obes Metab.

[5] Galván Casas C, Català A, Carretero Hernández G (2020). Classification of the cutaneous manifestations of COVID-19: a rapid prospective nationwide consensus study in Spain with 375 cases. Br J Dermatol.

[6] Attisani L, Pucci A, Luoni G (2021). COVID-19 and acute limb ischemia: a systematic review. J Cardiovasc Surg.

[7] Ocampo-Candiani J, Ramos-Cavazos CJ, Arellano-Mendoza MI (2021). International registry of dermatological manifestations secondary to COVID-19 infection in 347 Hispanic patients from 25 countries. Int J Dermatol.

[8] El-Mortada F, Landelouci K, Bertrand-Perron S (2023). Megakaryocytes possess a STING pathway that is transferred to platelets to potentiate activation. Life Sci Alliance.

[9] McGonagle D, Bridgewood C, Ramanan AV, Meaney JF, Watad A (2021). COVID-19 vasculitis and novel vasculitis mimics. Lancet Rheumatol.

[10] McGonagle D, Bridgewood C, Meaney JF (2021). A tricompartmental model of lung oxygenation disruption to explain pulmonary and systemic pathology in severe COVID-19. Lancet Respir Med.

[11] McGonagle D, O'Donnell JS, Sharif K, Emery P, Bridgewood C (2020). Immune mechanisms of pulmonary intravascular coagulopathy in COVID-19 pneumonia. Lancet Rheumatol.

[12] Middleton EA, He XY, Denorme F (2020). Neutrophil extracellular traps contribute to immunothrombosis in COVID-19 acute respiratory distress syndrome. Blood.

[13] Bettiol A, Alibaz-Oner F, Direskeneli H (2023). Vascular Behçet syndrome: from pathogenesis to treatment. Nat Rev Rheumatol.

[14] Szolnoky G (2020). Sulodexide may be a real alternative to low molecular weight heparins in the prevention of COVID-19 induced vascular complications. Dermatol Ther.

[15] Kapur A, Sharma M, Sageena G (2022). Therapeutic potential of N-acetyl cysteine during COVID-19 epoch. World J Virol.

[16] Zhang Z, Zheng Y, Niu Z (2021). SARS-CoV-2 spike protein dictates syncytium-mediated lymphocyte elimination. Cell Death Differ.

[17] Chamardani TM, Amiritavassoli S (2022). Inhibition of NETosis for treatment purposes: friend or foe?. Mol Cell Biochem.

